# Epidemiological profile and lethality of visceral leishmaniasis/human immunodeficiency virus co-infection in an endemic area in Northeast Brazil

**DOI:** 10.1590/0037-8682-0795-2020

**Published:** 2021-04-12

**Authors:** Carolina Angélica Libório Machado, Anaiá da Paixão Sevá, Arianna Araujo Falcão Andrade e Silva, Mauricio Claudio Horta

**Affiliations:** 1 Universidade Federal Rural de Pernambuco, Pós-Graduação em Biociência Animal, Recife, PE, Brasil.; 2 Universidade Estadual de Santa Cruz, Departamento de Ciências Agrárias e Ambientais, Ilhéus, BA, Brasil.; 3 Secretaria de Saúde do Estado de Pernambuco, Diretoria Geral de Vigilância de Doenças Negligenciadas e Sexualmente Transmissíveis, Recife, PE, Brasil.; 4 Universidade Federal do Vale do São Francisco, Petrolina, PE, Brasil.

**Keywords:** Pernambuco, AIDS, Leishmania, Epidemiology, Public health

## Abstract

**INTRODUCTION:**

The association of visceral leishmaniasis (VL) and human immunodeficiency virus (HIV) infection is a concern worldwide, and this co-infection is linked to increased lethality. The Northeast is the region that mostly reports cases of VL in Brazil. The knowledge of risk factors associated with VL/HIV co-infection and its impact on lethality is extremely important.

**METHODS:**

The present study analyzed the epidemiologic features of cases with VL/HIV co-infection in the state of Pernambuco, Northeast of Brazil, from 2014 to 2018.

**RESULTS:**

There were 858 and 11,514 reported cases of VL and HIV infection, respectively. The average incidences of VL and HIV infection were 1.82 and 24.4/100,000 inhabitants, respectively. Of all reported cases of VL, 4.9% (42/858) also had HIV infection. There was an inverse spatial association between VL and HIV infection incidences. The lethality rates of VL, HIV infection, and co-infection were 9.9%, 26.1%, and 16.6%, respectively. Most of the patients were males and lived in urban areas. The cases of VL mostly occurred in children aged below 10 years, whereas the cases of HIV infection and VL/HIV co-infection were primarily observed in adults between 20 years and 39 years old.

**CONCLUSIONS:**

We defined the profile and areas with most cases of co-infection and found that the lethality of VL with co-infection increased in the current period. These findings contribute to applying efforts with a greater focus in these identified populations to prevent future deaths.

## INTRODUCTION

Visceral leishmaniasis (VL) is a neglected parasitic disease that affects 76 countries worldwide. In the American continent, VL is considered endemic to 12 countries, with Brazil accounting for 96% of cases^1^. The disease is caused by protozoans of the genus *Leishmania.* In Brazil, the disease is caused by *Leishmania infantum* ^2^ *.* The agent is transmitted through bites of sandflies of the genus *Lutzomyia* ^*3*^.

Until 1990, more than 90% of Brazil's VL cases occurred in the Northeast region^4^, and from 1990 to 2018, 64.2% of cases were reported in this region, with more than 91,000 cases in the country^5^. The reduction during the latter period was due to a change in the disease pattern of VL once it spread to other regions of Brazil.

This zoonosis is considered a growing public health problem with a wide geographic distribution. VL tends to spread to non-endemic regions, and it is highly lethal if not treated^6^. Early treatment can lower its mortality rate, and it is one of the recommendations of the Brazilian Ministry of Health for the prevention and control of VL^4^.

Knowledge of the factors associated with VL infection is critical for planning control and prevention in endemic areas or areas at risk of infection. Environmental and socioeconomic factors, such as vegetation cover, weather, poverty, and living space, influence the occurrence of infection^7^, while the host's immunity directly influences the development of the disease^8^.

The association of VL and human immunodeficiency virus (HIV) is a global concern, and this co-infection is linked to aggravating the symptoms of the disease and increasing the lethality (proportion of cases that results in death within a specified period of time^9^) of both diseases^10^. A specific interaction between leishmania and HIV at the cellular level is responsible for affecting the course of infection for each pathogen. Macrophages and dendritic cells have been observed to lead to such interactions. The involvement of other cells may explain the clinical development observed in co-infected patients^11^. The number of people with VL/HIV co-infection has been increasing in Brazil; the lethality rate of VL/HIV co-infection is three times higher than that of VL isolated^12^.

In the past decade, the Northeast region has reported over 96,000 cases of Acquired Immunodeficiency Syndrome (AIDS), the disease caused by HIV. The state of Pernambuco has the second-highest number of cases in the region, behind Bahia^13^⁠. In addition, most reported VL cases are in this region^14^, and reports of both diseases are considered high in areas with high poverty rates^12^.

The knowledge of the areas with cases of VL/HIV co-infection and both diseases individually can contribute to making quick diagnosis and treatment; thus, reducing the lethality of both diseases. Similarly, the knowledge of factors associated with co-infection is fundamental in planning for the prevention and control of cases. Thus, this study aimed to analyze the epidemiological profile of reported cases of this co-infection in the state of Pernambuco, in the Northeast region of Brazil, from 2014 to 2018, being a purely descriptive observational analysis of secondary data.

## METHODOLOGY

### Study area

**FIGURE 1:**
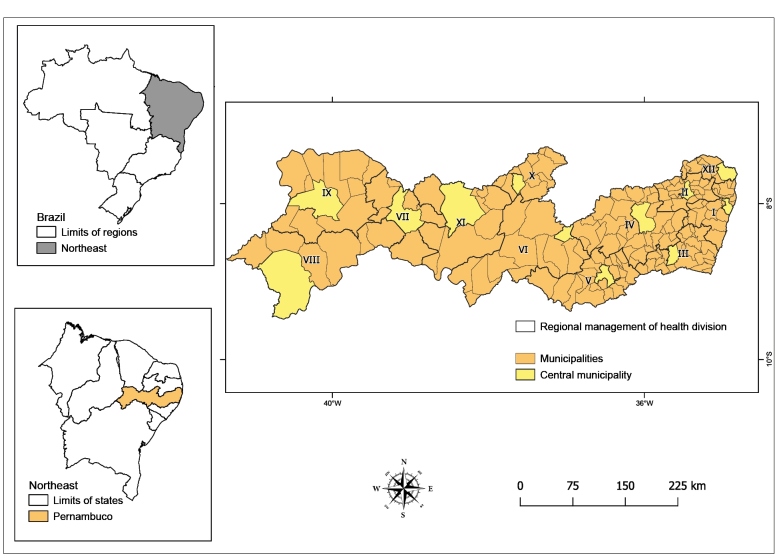
Regional management of health division of Pernambuco state at the Northeast region of Brazil.

Located in the eastern center of the Northeast region of Brazil, Pernambuco has an area of 98,311 Km^2^. It has the second largest economy of the region and a medium Human Development Index (HDI) score (0.673). It is divided into 12 health management regions with a central municipality in each region: I. Recife, II. Limoeiro, III. Palmares, IV. Caruaru, V. Garanhuns, VI. Arcoverde, VII. Salgueiro, VIII. Petrolina, IX. Ouricuri, X. Afogados da Ingazeira, XI. Serra Talhada, and XII. Goiana (Figure 1)^15^ ^,^ ^16^.

### btaining Data

Data of reported cases of VL and HIV infection, between 2014 to 2018, were obtained directly from the Pernambuco State Department of Health. They were analyzed according to the municipality of residence, age (< 10 years old, 10-19 years old, 20-39 years old, 40-59 years old, or > 60 years old), sex (male or female), level of education (incomplete basic education, complete basic education, incomplete elementary school, complete elementary school, incomplete high school, complete high school, incomplete university undergraduate, complete university undergraduate, or ignored), area of residence (urban or rural) and outcome (cure, transfer, abandonment of treatment, death, or ignored). "Ignored" was used for missing information. Incidences and death rates of both diseases and co-infection were calculated according to the estimated population from DATASUS (Department of Informatics of the Health System).

### Data analysis

The descriptive analysis considered frequencies of the analyzed variables and their confidence intervals at 95% (according to Newcombe, 1998)^17^, excluding ignored answers, by using Microsoft Office Excel®. Statistical analysis (chi-square/Fisher exact tests) was performed using R Studio (version 3.6.1). The spatial distribution cases in the municipalities were performed using QGIS software (version 2.18).

Spatial correlation analyses were performed between the municipality incidences of both VL and HIV infection separately and each one with co-infection, all in pairs, using Bivariate Moran's statistics. This analysis considered the correlation between one variable in the municipality and other variables of all municipalities surrounding it. In other words, the association matrix between municipalities is called Queen Contiguity of the first-order matrix. Thus, this correlation process was carried out for each municipality in the entire state, resulting in a value for each called the Local Moran Index (LISA). To validate the result of these correlations, by checking if they are random or not, 999 permutations of the same analysis were performed, generating a *pseudo* p-value. A global Moran's Index was also generated, which considered the average difference of LISA of all municipalities. There was a significant correlation when p*<*0.05 for p-values of LISA and for *pseudo* p-values. Analyses and the LISA map were performed using GeoDa (version 1.14.0).

## RESULTS

From 2014 to 2018, , 858 and 11,514 autochthonous cases of VL and HIV infection, respectively, were reported in Pernambuco. The average incidences of VL and HIV infection were 1.8 and 24.4 per 100,000 inhabitants, respectively. Of all reported cases of VL, 4.9% (42/858) also had HIV infection, 67.2% (577) were HIV negative, and 27.8% (239) were listed as "ignored."

**FIGURE 2:**
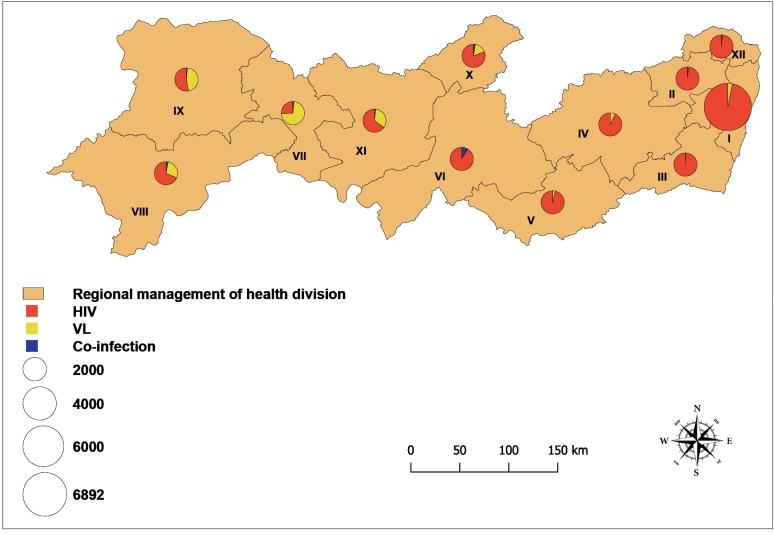
Numbers of VL, HIV infection, and VL/HIV co-infection cases by regional management of health division in Pernambuco, 2014-2018. **VL:** visceral leishmaniasis; **HIV:** human immunodeficiency virus.

The capital of the state (Recife) is located in region I, which has the highest number of reported cases of VL (*n* = 238/858, 27.7%) and HIV infection (*n* = 7,913/11,514, 68.7%). Region VIII had the highest number of VL/HIV co-infection cases ( *n* = 15/42, 35.7%), with the municipality of Petrolina standing out. The approximate number and proportion of cases distributed by region are shown in Figure 2.

**FIGURE 3:**
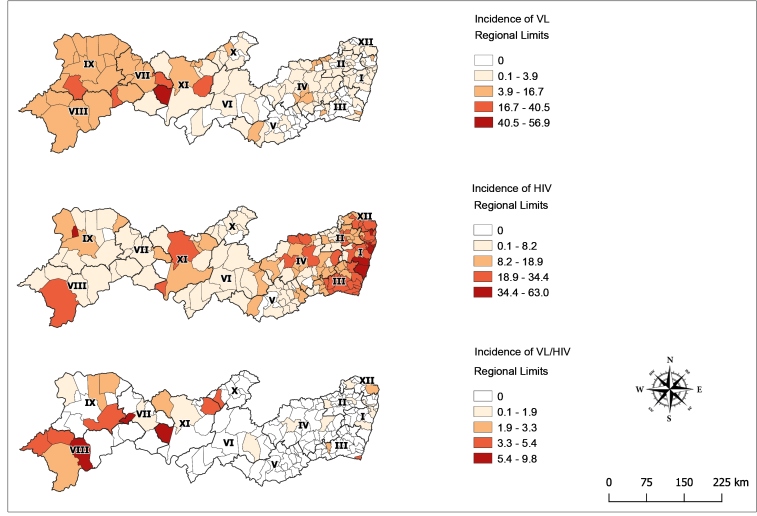
Incidence of VL, HIV infection, and VL/HIV co-infection in Pernambuco state, 2014-2018. **VL:** visceral leishmaniasis; **HIV:** human immunodeficiency virus.

Petrolina was the municipality with the most reported cases of VL, accounting for 8.7% (75/858) of cases during the entire study period. Of these cases, 14.6% (11/75) were HIV positive, making it the municipality with the highest VL/HIV co-infection cases. Considering only HIV case reports, Recife (region I) was the municipality with the most reported cases (*n* = 3,443, 29.9%), followed by Jaboatão dos Guararapes, Olinda (both in region I), Caruaru (region IV), and Petrolina (region VIII). The highest VL incidence was observed in the health management regions VII, VIII, IX, and XI and co-infection with HIV in regions I, III, and XII (Figure 3).

**FIGURE 4:**
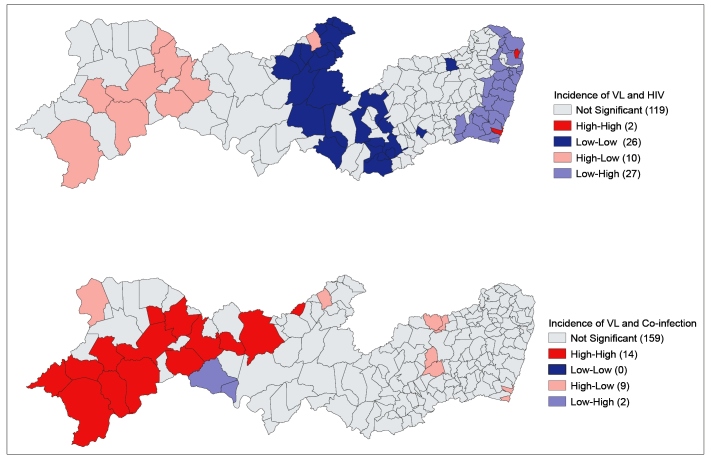
Cluster map of Local Indicators of Spatial Association between VL and HIV infection and VL and VL/HIV co-infection in the municipalities of Pernambuco state.

Legend: The legends represent correlations between incidences of both VL and HIV infection and VL and VL/HIV co-infection, wherein the groups the 1^st^ level (high or low) represents the VL incidence and the 2^nd^ represents the HIV infection incidence (upper map) and co-infection (bottom map). Not significant are municipalities without significance in autocorrelation (p < 0.05). Number in parenthesis refers to the municipalities in each group. VL: visceral leishmaniasis; HIV: human immunodeficiency virus.

Comparing diseases in municipalities, a significant spatial correlation, with a predominantly inverse association, was found between the incidences of VL with HIV infection (global Moran's index = -0.126 and pseudo p-value = 0.001) in 35.3% (65) of municipalities with p values < 0.05. There was a high incidence of HIV infection and low incidence of VL in the coastal region, representing 14.6% (27) of the state's municipalities (Figure 4, in light blue). The opposite was observed in the west of the state, where 5.4% (10) of municipalities had a high incidence of VL and low incidence of HIV infection (in light red). In the center of the state, 14.1% (26) of municipalities had a high incidence of both diseases (in dark blue). The spatial correlations of VL and HIV infection with VL/HIV co-infection were also significant (global Moran’s index = 0.258 and pseudo p-value = 0.001) in 13.6% (25) of municipalities (p < 0.05), showing a direct association with both values. Spatial correlation for HIV infection and VL/HIV co-infection were both insignificant.

The lethality rate in the study period was 10.5% (*n* = 78/741) for VL and 26.1% (*n* = 3,009/11,472) for HIV infection. The lethality rate was 18.4% (*n* = 7/38) among patients with VL/HIV co-infection. Among VL cases, those with HIV had a higher lethality rate than those without HIV; however, without statistical significance (*p* > 0.05). Considering deaths of VL and of HIV, 8.2% (7/85) and 0.2% (7/3016) were caused by co-infection, respectively.

Regarding the profile of the reported cases of VL, 41.2% (*n* = 354/858) were in children less than 10 years old, 65.2% (*n* = 559/858) were males and 55.3% (*n* = 465/858) were from urban areas (Table 1). Analyzing the level of education, in 45.7% (*n* = 305/667) of the reports, the responses were "not applicable," which refers to the preschool period. Regarding the evolution of cases, 83.8% (*n =* 621/741) were cured, 11.5% (*n =* 85/741) died, and 13.6% (*n =* 117/858) had their outcome ignored.

**TABLE 1:** Profile of reported cases of visceral leishmaniasis (VL), human immunodeficiency virus (HIV) infection and co-infection VL/HIV of Pernambuco State, 2014-2018.

Variables	Categories	VL		HIV		VL/HIV	
		N	Freq (%); CI 95%	N	Freq (%); CI 95%	N	Freq (%); CI 95%
Sex	Males	559	65.2; 61.9 - 68.2	7,726	67.1; 66.2 - 67.9	32	76.2; 61.4 - 86.5
	Females	299	34.8; 31.7 - 38.1	3,788	32,9; 32.0 - 33.7	10	23.8; 13.4 - 38.5
Age	< 10 years old	354	41.2; 38.0 - 44.5	41	0.3; 0.2 - 0.4	5	11.9; 5.1 - 25.0
	10-19 years old	96	11.2; 9.2 - 13.4	706	6.1; 5.7 - 6.5	2	4.8; 1.3 - 15.7
	20-39 years old	210	24.5; 21.7 - 27.4	7,438	64.6; 63.7 - 65.4	16	38.1; 25.0 - 53.1
	40-59 years old	130	15.1; 12.9 - 17.7	3,003	26.1; 25.2 - 26.8	15	35.7; 22.9 - 50.8
	>60 years old	68	8.0; 6.3 - 9.9	326	2.9; 2.5 - 3.1	4	9.5; 3.7 - 22.0
Education Level	Not applicable	305	45.7; 41.9 - 49.5	-		5	20.0; 8.0 - 39.1
	Illiterate	48	7.2; 5.4 - 9.4	286	3.4; 3.0 -3.8	2	8.0; 2.0 - 24.9
	Incomplete primary	79	11.8; 9.6 - 14.5	850	10.2; 9.6 - 10.9	7	28.0; 14.2 - 47.5
	Complete primary	18	2.7; 1.7 - 4.2	599	7.2; 6.7 - 7.8	1	4.0; 0.7 - 19.5
	Incomplete elementary school	110	16.5; 13.8 - 19.5	1,433	17.3; 16.5 - 18.1	5	20.0; 8.0 - 39.1
	Complete elementary school	38	5.7; 4.1 - 7.7	731	8.8; 8.2 - 9.4	1	4.0; 0.7 - 19.5
	Incomplete high school	26	3.9; 2.6 - 5.6	788	9.5; 8.9 - 10.1	3	12.0; 4.1 - 29.9
	Complete high school	35	5.2; 3.8 - 7.2	2,208	26.6; 25.7 - 27.6	-	
	Incomplete university graduation	2	0.3; 0.8 - 1.0	645	7.9; 7.2 - 8.4	-	
	Complete university graduation	6	1.0; 0.4 - 1.9	753	9.1; 8.5 -9.7	1	4.0; 0.7 - 19.5
	Ignored	191		3,172		17	
Residence area	Urban	465	55.3; 51.9 - 58.6	-		33	82.5; 68.0 - 91.2
	Rural	372	44.2; 40.9 - 47.6	-		7	17.5; 8.7 - 31.9
	Peri-urban	4	0.5; 0.09 - 1.2	-		-	
	Ignored	17		-		2	
Outcome	Cure	621	83.8; 80.9 - 86.2	-		27	71.0; 52.2 - 83.0
	Death	85	11.5; 9.3 - 13.9	2,805	24.3; 23.5 - 25.1	7	18.4; 9.2 - 33.4
	Transfer	32	4.3; 3.0 - 6.0	-		3	8.0; 2.7 - 20.8
	Abandonment	3	0.4; 0.1 - 1.1	-		1	2.6; 0.05 -13.5
	Ignored	117		-		4	

Of the reports of HIV infection, 67.1% (*n =* 7,726/11,514) were males, 64.6% (*n =* 7,438/11,514) were between 20 years and 39 years old, and 27.9% (*n =* 3,172/11,514) were listed as “ignored”. Regarding the level of education, 19.2% (*n =* 2,208/11,514) had completed high school.

When considering only the patients with VL/HIV co-infection, 76.2% (*n =* 32/42) were males, 38.1% (*n =* 16/42) were between 20 years and 39 years old, 35.7% (*n =* 15/42) were between 40 years and 59 years old, and 82.5% (*n =* 33/42) were from urban areas. A total of 40.4% (*n =* 17/42) of the reports listed "ignored" regarding the level of education, and 20% (*n =* 5/25) of the reports did not complete basic education or were listed as "not applicable." 

Concerning the profile of the co-infected patients who died, 71.4% (*n =* 5/7) were males, 57.1% (*n* = 4/7) were between 20 years and 39 years old, 71.4% (*n =* 5/7) were from urban areas, and 42.8% (*n =* 3/7) listed “ignored” in regards to level of education. The top therapeutic choices for co-infected patients were pentavalent antimonial drugs (38%, *n =* 16/42), amphotericin B (28.6%, *n =* 12/42), and liposomal amphotericin B (14.3%, *n =* 6/42). Of the 7 co-infected patients who died, 3 were treated with pentavalent antimonial, two with amphotericin B, and two were not treated. No patients were treated with liposomal amphotericin B. Overall, 60.9% (*n =* 27/42) of patients were cured, and 16.6% (*n =* 7/42) died from VL or other causes.

## DISCUSSION

Lethality of VL increases by 7.9% when associated with HIV infection; previous corroborating studies found an increase in lethality ranging from 4.6% to 16.6% in co-infected patients from the Northeast (Rio Grande do Norte) and Southeast (Minas Gerais) regions^18^ ^-^ ^20^. This is explained by the fact that the two infectious diseases may cause infection and multiply in lymphoid cells and act by increasing the capacity for viral and parasite replication, favoring the clinical evolution of both diseases^21^ ^,^ ^22^.

Endemic in 35 countries, VL/HIV co-infection is a global concern. The presence of HIV leads to severe forms of VL, which are difficult to control and manage. Furthermore, people with HIV infection are more likely to get VL, which increases virus replication and the clinical evolution of AIDS. Therefore, this co-infection warrants concern and must be investigated and adequately monitored^23^.

Associated with the pathogenesis of diseases, the choice of treatment influences the lethality in patients with co-infection. The choice of derivative antimonials as a therapeutic protocol is directly linked to the severity of clinical evolution and increasing lethality^19^. The World Health Organization (WHO) recommends that patients with co-infection be treated with liposomal amphotericin B^11^.

In Brazil, the first treatment option is pentavalent antimonial drugs. However, due to its high toxicity and side effects, in some cases, such as in pregnant women, children under the age of one, the elderly, and patients with comorbidities and co-infection with HIV, other medications, such as amphotericin B and liposomal amphotericin B, are prescribed^24^.

It was observed that of the patients who died, none was treated according to the WHO protocol. Overall, lethality in patients with co-infection may be related to the predisposition of infection or associated with other opportunistic infections^18^.

Another important factor to note in this study is the high presence of isolated cases of both HIV infection and VL in urban areas. This favors VL/HIV co-infection and the establishment of co-infection cases in urban areas, as a high percentage of co-infection was also observed in urban areas. The urbanization of VL in the 1980s and the presence of the circulating virus in this same population increased the risks of co-infection^4^.

Despite the two diseases, both isolated and concomitantly, being found mainly in urban areas, the relationship of co-infection with both LV and HIV infection was not significant, and there was an inverse spatial autocorrelation in the distribution of each individually in the entire state. It was observed that VL was concentrated in the western region of the state, HIV infection was concentrated on the coast, and the central region had a low incidence of both. VL has a history of high incidence in the Northeast interior due to climatic and socioeconomic factors^7^ ^,^ ^25^. The areas with the highest exposure to HIV in the Northeast are located close to the coast and are related to sexual transmission, with evidence of being enhanced by "sex tourism."^26^

From 2014 to 2018, there were 167,741 reported cases of HIV infection in Brazil, 20.5% (34,414) of which were in the Northeast region^27^. In Pernambuco, they steadily increased until 2017, when a reduction started. However, it is important to note that HIV infections were only compulsorily reported from 2014; before that, only AIDS cases were monitored. This demonstrates that the system is relatively new, and divergences or reporting failures may occur, which would explain the oscillation of the values.

Regarding VL infection in the study period, Pernambuco maintained an average of 162 cases per year, with an increase in 2018 by 208 cases. The average number of VL/HIV co-infection cases remained similar, except in 2015, when only one case was reported. However, it is essential to note that 27.8% (239/858) of the cases were not tested for HIV and listed "ignored" to this question. Even with an increase in the percentage of deaths, the lack of knowledge of co-infection in 27% of cases hindered the assessment of the actual situation.

Region I was responsible for the highest VL and HIV infection reports separately. It is important to remember that this region includes the metropolitan region of the capital Recife, which has the highest population of the entire state. Evaluating patients with co-infection, the region with the highest number of reports was Region VIII, whose central city is Petrolina.

Petrolina is the municipality that reported the most cases of VL and VL/HIV co-infection in the whole state in the study period, and has historically reported the largest numbers of cases in the state^14^ ^,^ ^28^. Petrolina is far from the coast and the state's capital, it is the second-most populous municipality of the state, excluding the metropolitan region^29^. It is an important center of fruit production for the country, being a place with a high flow of people from various areas, contributing to the high number of VL cases^30^.

Concerning the profile of patients with VL, HIV infection, and VL/HIV co-infection, most patients were male. For VL, the majority of cases were in children below the age of 10. In contrast, most reported cases of HIV infection and VL/HIV co-infection were in adults aged between 20 years and 39 years. This corroborates data analyzed throughout the world and in Brazil, where most cases of co-infection are in young male adults^11^ ^,^ ^31^ ^-^ ^33^.

Regarding the level of education, "ignored" was the most frequently found response in the reported cases of VL, HIV infection, and VL/HIV co-infection. The fact that adequate information about the level of education was not available for many patients interfered with the analysis of this risk variable.

In conclusion, this study revealed that VL/HIV co-infection in Pernambuco increases lethality in young, adult male patients who did not receive liposomal amphotericin B as a treatment. It is necessary to appropriately report patient data, screen all patients with HIV infection and VL for both infections, and promptly follow the recommended approach for treating patients with co-infection.
